# 超高效液相色谱-高分辨质谱联用结合整合过滤策略全面分析茶树花中化学成分

**DOI:** 10.3724/SP.J.1123.2021.07015

**Published:** 2022-03-08

**Authors:** Sichen HUANG, Hongpeng ZHAO, Yongdan HU, Dabing REN, Lunzhao YI

**Affiliations:** 昆明理工大学农业与食品学院, 云南 昆明 650500; College of Agriculture and Food, Kunming University of Science and Technology, Kunming 650500, China; 昆明理工大学农业与食品学院, 云南 昆明 650500; College of Agriculture and Food, Kunming University of Science and Technology, Kunming 650500, China; 昆明理工大学农业与食品学院, 云南 昆明 650500; College of Agriculture and Food, Kunming University of Science and Technology, Kunming 650500, China; 昆明理工大学农业与食品学院, 云南 昆明 650500; College of Agriculture and Food, Kunming University of Science and Technology, Kunming 650500, China; 昆明理工大学农业与食品学院, 云南 昆明 650500; College of Agriculture and Food, Kunming University of Science and Technology, Kunming 650500, China

**Keywords:** 超高效液相色谱-高分辨质谱, 整合过滤策略, 化学成分, 茶树花, ultra-performance liquid chromatography-high resolution mass spectrometry (UPLC-HRMS), integrated filtering strategy (IFS), chemical constituents, tea flower

## Abstract

茶树花与茶鲜叶同为茶树的生物产出,但茶树花往往被视为茶叶生产过程中的废物被舍弃,造成了茶树花资源的极大浪费。目前对于茶树花中化学成分的分析主要集中在氨基酸、茶多酚等单一类型化学成分上,对于茶树花中多类化学成分的同时分析仍鲜见报道。研究者对于茶树花中所含化学成分的种类和含量不完全清楚,成为制约茶树花深度开发与利用的重要原因。该研究采用超高效液相色谱-高分辨质谱联用技术(UPLC-HRMS)对茶树花中的化学成分进行检测,结合氮规则过滤(NRF)、质量亏损过滤(MDF)和诊断碎片离子过滤(DFIF)的整合过滤策略(IFS)筛选目标化学成分的特征质谱,并利用化学成分的色谱保留时间、一级质谱、二级质谱等信息对化学成分进行定性分析。共定性出茶树花中6大类共137种化学成分,包括3种生物碱、38种黄酮、31种酚酸及其衍生物、37种儿茶素及其衍生物、18种氨基酸以及10种其他类成分。采用内标法对所有定性出的137种化学成分进行定量分析,结果表明,茶树花中6类化学成分含量从高到低依次为氨基酸(9371.42 μg/g)、儿茶素及其衍生物(9068.43 μg/g)、酚酸及其衍生物(8696.92 μg/g)、生物碱(4392.52 μg/g)、黄酮(1192.88 μg/g)、其他类成分(139.94 μg/g)。该研究采用质量控制样本评价仪器的稳定性和检测数据的重复性,9种代表性化学成分的相对标准偏差在2.11%~12.17%范围内,表明仪器的稳定性和检测数据重复性良好。同时,选取绿原酸类成分以及糖基化槲皮素类成分作为代表性成分,说明了整合过滤策略提取目标类型化学成分的全过程。该研究全面揭示了茶树花中化学成分的种类和含量,可为茶树花的深度开发和利用提供有价值的信息和数据参考。

## Abstract (en)

Tea flowers and fresh tea leaves are biological products of tea, but tea flower is often regarded as waste during tea production, resulting in notable waste of tea flower resources. At present, analysis of the chemical components in tea flowers focuses on single types of chemical components such as amino acids and tea polyphenols, and there are only a few reports on the simultaneous analysis of numerous chemical components in tea flowers. Researchers are not completely clear about the types and amounts of the chemical components in tea flowers; this has hindered the in-depth development and effective utilization of tea flowers. In this study, ultra-performance liquid chromatography-high resolution mass spectrometry (UPLC-HRMS) was used to detect the chemical constituents of tea flowers. This technique was combined with the integrated filtering strategy (IFS) of nitrogen rule filtering (NRF), mass defect filtering (MDF), and diagnostic fragment ion filtering (DFIF) for screening the characteristic mass spectra of the target chemical components. Furthermore, the chemical constituents of tea flowers were annotated with information about the retention time, MS fragmentation, and MS/MS fragmentation. All the qualitative chemical components were divided into six categories with a total of 137 chemical constituents, including 3 alkaloids, 38 flavonoids, 31 phenolic acids and their derivatives, 37 catechins and their derivatives, 18 amino acids, and 10 other components. The internal standard method was used to quantify all the qualitative chemical components. The quantitative results showed that the amounts of the six kinds of chemical components in tea flowers were as follows: amino acids, 9371.42 μg/g; catechins and their derivatives, 9068.43 μg/g; phenolic acids and their derivatives, 8696.92 μg/g; alkaloids, 4392.52 μg/g; flavonoids, 1192.88 μg/g; and others, 139.94 μg/g. Quality control samples were used to evaluate the stability of the instrument and the repeatability of the tested data. Nine representative chemical components with high, medium, and low contents in tea were selected, and the relative standard deviation (RSD) of the results was used to evaluate the repeatability of the data. These nine chemical constituents are selected from amino acids, alkaloids, flavonoids, phenolic acids and their derivatives, catechins and their derivatives, and other components, and the response intensities were different. The relative standard deviations of the nine chemical components were in the range of 2.11% to 12.17%. The above results demonstrated the good stability of the instrument and excellent repeatability of the test data. Chlorogenic acid components (CGAs) and glycosylated quercetin components (GQs) were used as two representative components to explain the entire process of extracting the target compounds by IFS. CGAs comprise a class of special esters formed by the esterification of cinnamic acid derivatives with quinic acid as the parent structure. The most common cinnamic acid derivatives are *p*-coumaric acid, caffeic acid, and ferulic acid. On the one hand, according to the above information and the different positions and degree of quinic acid esterification, the CGAs were structurally classified as monosubstituted CGAs (Mono-CGAs), disubstituted CGAs (Di-CGAs), and trisubstituted CGAs (Tri-CGAs), and three different mass defect filtering windows were set. Therefore, 751 possible target components were selected from 3537 mass spectra in accordance with the nitrogen rule. On the other hand, 22 target components in accordance with the nitrogen rule were obtained by further screening the *m/z* 191.0551 ion as the diagnostic fragment ion of the CGAs. Combining the overall analytical data with the above mass defect filtering and diagnostic fragment ion filtering screening results, nine target CGAs were selected and characterized based on the MS information. This study reveals the types and amounts of the chemical components accumulated in tea flowers, thus providing valuable information and serving as data reference for the in-depth development and effective utilization of tea flowers.

茶树花与茶鲜叶同是茶树(*Camellia sinensis* (L.) O. Kuntze)的生物产出。茶树花一般于每年5月份开始花芽分化,开花时间因茶树品种与种植地区而异,寿命一般为2天,结实率较低,具有“寿命短、花期长、结实少”的特点^[1,2]^。我国茶树花资源十分丰富,全国茶区每年可产茶树花300多万吨^[3]^。茶树花能够被加工成多种产品,例如茶树花酒^[4]^、茶树花香皂^[5]^等,也可以经干燥后直接冲泡饮用^[6]^。但长期以来,人们只是采摘茶树的鲜嫩芽叶制茶,对于茶树花,绝大部分任其自生自灭,甚至通过人为方法去除茶树花,以达到茶叶高产的目的,这对茶树花资源是一种极大浪费^[1,7]^。

近年来随着研究的不断深入,茶树花的内含物质被不断发现^[8,9]^。据研究报道,茶树花含有与茶叶相似的化学成分,包括多酚、生物碱、酚酸、多糖和芳香化学成分等,对人体具有抗氧化、抗糖尿病、抗高血脂、抗高血糖、减肥等功效^[10]^。Chen等^[11]^将茶树花中粗蛋白酶分离、提取和纯化,并利用液相色谱-串联质谱法进行了分析,首次发现茶树花中含有对茶叶蛋白质具有较强水解能力的蛋白酶并能够使茶汤当中的氨基酸总量大大增加;徐人杰等^[3]^采用高效液相色谱法对茶树花中可溶性糖、儿茶素和游离氨基酸的含量进行了检测,结果显示,茶树花中含有儿茶素和茶氨酸等成分,并且发现茶氨酸含量占总游离氨基酸的50%左右。然而,目前对于茶树花中化学成分的分析主要集中在氨基酸、茶多酚等单一类型化学成分上,对于多类化学成分的同时分析仍鲜见报道。

超高效液相色谱-高分辨质谱联用技术(ultra-performance liquid chromatography-high resolution mass spectrometry, UPLC-HRMS)是一种强大的分析工具,具有高通量、高选择性和高灵敏度的特点,在多类化学成分的同时分析检测方面得到了越来越广泛的应用^[12,13,14]^。然而,目前仍鲜见利用UPLC-HRMS对茶树花中化学成分进行分析的报道。此外,UPLC-HRMS数据非常复杂,含有大量的背景噪声和干扰离子,给后续化学成分的定性分析带来了许多困难^[15]^。为了有效去除干扰离子,提取目标成分的质谱特征,目前已有研究提出了多种质谱过滤方法,包括质量亏损过滤(mass defect filtering, MDF)^[16]^、氮规则过滤(nitrogen rule filtering, NRF)^[15]^、中性丢失过滤(neutral loss filtering, NLF)^[17]^和诊断碎片离子过滤(diagnostic fragment ion filtering, DFIF)^[18]^等。质量亏损是一种元素或者化学成分的精确质量与其最接近整数值之间的差值。质量亏损过滤可以排除目标质量范围外的化学成分,降低质谱解析的复杂度^[17]^。氮规则是指不含氮或含偶数氮的有机物的相对分子质量为偶数,含奇数氮的有机物的相对分子质量为奇数,氮规则过滤可基于该规则对质谱数据进行初步过滤^[19]^。中性丢失是指物质在裂解过程中,失去的一些不带电荷的基团。诊断碎片离子是指可表征化学成分结构特征的质谱碎片离子,具有相同或相似骨架的化学成分在相同的碰撞电压下会有相似的质谱裂解,产生相似的质谱碎片。中性丢失过滤和诊断碎片离子过滤可以快速识别具有相同或相似骨架结构或具有相同官能团的化学成分^[18]^。然而,单一过滤策略很难避免定性分析过程中的假阳性和假阴性。与单一过滤策略相比,整合过滤策略(integrated filtering strategy, IFS)融合了多种质谱过滤方法,例如MDF、NRF以及DFIF相结合,在去除干扰离子和检测目标成分方面已被证实更有效^[15,18]^。本研究以茶树花作为研究对象,结合UPLC-HRMS技术和IFS,全面分析茶树花中化学成分的组成和含量,为茶树花的综合利用和开发提供有价值的信息。

## 1 实验部分

### 1.1 仪器、试剂与材料

UltiMate 3000超高效液相色谱、Q Exactive台式四极杆-轨道阱高分辨质谱(美国Thermo Scientific公司); TGL-16M高速离心机(湖南湘仪实验室仪器开发有限公司); SK5200GT超声波清洗器(上海科导超声仪有限公司); Milli-Q A10超纯水机(美国Millipore公司); AQ-180E磨粉机(慈溪市欧耐电器有限公司)。

甲醇、乙腈(质谱级,德国Merck公司);甲酸(质谱级,美国Honeywell公司);标准品共计78种,包括生物碱3种、氨基酸18种、儿茶素及其衍生物13种、酚酸及其衍生物13种、黄酮24种、其他类成分7种,纯度均在97%及以上。

茶树花样品7份,均于2019年4月采自云南省德宏州盈江县油松岭。所有样品经冷冻干燥以及打粉,并过60目筛后放置于-20 ℃冰箱中保存备用。

### 1.2 色谱与质谱条件

色谱条件 色谱柱:Waters ACQUITY UPLC^®^ HSS C_18_柱(100 mm×2.1 mm, 1.8 μm);柱温:35 ℃;流动相:A为乙腈溶液,B为0.1% (v/v)甲酸溶液;流速0.2 mL/min;进样量:1 μL。洗脱梯度:0~3 min, 95%B~93%B; 3~4 min, 93%B~90%B; 4~8 min, 90%B; 8~15 min, 90%B~60%B; 15~18 min, 60%B~50%B; 18~20 min, 50%B~95%B; 20~25 min, 95%B。

质谱条件 电喷雾离子源(ESI),采用正、负离子模式检测;喷雾电压:3500 V(+), 4000 V(-);雾化温度:300 ℃;雾化气(鞘气)流速:30 L/min;辅助气流速:10 L/min;传输毛细管温度:320 ℃;扫描模式:全扫描(full scan),分辨率35000;源内诱导裂解电压(in-source CID): 0 eV;数据依赖二级扫描(ddMS^2^),分辨率17500;高能碰撞诱导电压(HCD stepped): 25、35、45 eV。

### 1.3 样品前处理

准确称取样品0.100 g,加入内标溶液(0.1 g/L葛根素和磺胺醋酰)各0.1 mL以及1 mL 70%(v/v)甲醇水溶液,涡旋混合均匀后进行超声波辅助提取(40 ℃, 15 min)。随后将提取液进行离心(10000 r/min, 5 min)并取上清液于2 mL容量瓶中。之后分别取两次0.5 mL 70%(v/v)甲醇水溶液再重复两次上述提取步骤。最后用70%(v/v)甲醇水溶液定容并取1 mL样品提取液过0.22 μm微孔滤膜于进样瓶中,保存至4 ℃冰箱备用。每个样品做3次平行。

### 1.4 QC样本制备

取7种茶树花样品提取液各50 μL于棕色瓶中,涡旋混合均匀后用0.22 μm微孔滤膜过滤到进样瓶中作为质量控制样本(quality control, QC),保存至4 ℃冰箱备用。QC样本用于评价仪器稳定性和数据重复性。

### 1.5 定性定量分析

定性分析 采用Xcalibur 3.0软件分析UPLC-HRMS数据中化学成分的保留时间、一级质谱和二级质谱。对于目标类型化学成分,如绿原酸类成分(chlorogenic acid components, CGAs)和糖基化槲皮素类成分(glycosylated quercetin components, GQs)等,采用MZmine 2.38软件对原始UPLC-HRMS数据进行色谱峰构建、峰平滑及解卷积等处理,提取获得具有化学成分保留时间、质荷比和峰面积等信息的质谱峰列表。采用集合NRF、MDF和DFIF的IFS方法去除干扰质谱,获得目标类型化学成分的质谱特征。IFS能够以目标化学成分的诊断碎片离子、氮原子数量以及质量窗口等作为筛选条件对所得质谱数据进行过滤,大大提高筛选目标类型化学成分的效率,降低质谱数据解析的难度。在此基础上,对于有标准品的78种化学成分,通过与标准品的一级质谱、二级质谱数据及保留时间比对,从而实现定性。其他化学成分则通过数据库匹配,包括Tea Metabolome Database (TMDB)和Tea Plant Information Archive (TPIA),并结合参考文献中的一级质谱和二级质谱信息实现定性。

定量分析 采用内标法对各组分进行定量分析。内标物的选择不仅要尽可能与待测物质性质相似,而且要避免与上百个待测物质中的任意物质共流出,且在定量分析中需兼顾多类物质的含量区间。由于磺胺醋酰与葛根素的物理化学性质与茶树花中主要化学成分儿茶素类和黄酮类相似,并且在UPLC-HRMS分析中能够得到质谱响应强度适中且基线分离的色谱峰,因此磺胺醋酰和葛根素分别作为正离子以及负离子模式下的内标物。UPLC-HRMS数据通过MZmine 2.38软件进行分析,提取出化学成分的峰面积、保留时间、准分子离子质量,最后通过式(1)和(2)分别计算相对校正因子以及化学成分的含量。


(1) $F=\frac{A_{s} / C_{s}}{A_{r} / C_{r}}$

式中,*A_s_*:混合标准溶液中内标物的峰面积;*A_r_*:混合标准溶液中标准品的峰面积;*C_s_*:混合标准溶液中内标物的质量浓度,g/L; *C_r_*:混合标准溶液中标准品的质量浓度,g/L。


(2) $w_{i}=\frac{A_{i} m_{s}}{A_{k} m} F$

式中,*A_k_*:供试样品液中内标物的峰面积;*A_i_*:供试样品液中化学成分的峰面积;*m_s_*:供试样品液中内标物的质量,μg; *w_i_*:样品中待测化学成分的含量,μg/g; *m*:样品的质量,g; *F*:相对校正因子。

其余未用标准品定性的化学成分含量通过式(3)计算,所有的字母定义和式(2)一致:


(3) $w_{i}=\frac{A_{i} m_{s}}{A_{k} m}$

## 2 结果与讨论

### 2.1 仪器稳定性和数据重复性检验

为保证数据质量,采用QC样本数据评价仪器的稳定性和检测数据重复性。选取低含量代表性成分鸟苷、木犀草苷和L-组氨酸,中等含量代表性成分D-色氨酸、没食子酸和柠檬酸以及高含量代表性成分儿茶素、咖啡因和可可碱,以检测结果的相对标准偏差(relative standard deviation, RSD)评价数据重复性和仪器稳定性。以上9种化学成分的RSD值均在2.1%~12.2%范围内,表明数据重复性好、仪器稳定性高。这9种化学成分分属本研究中的6类化学成分并且响应强度也各有不同。

### 2.2 IFS提取目标类型化学成分的质谱特征

茶树花提取物总离子流图如图1所示。从图1可以看出,茶树花提取物中含有大量的化学成分。采用MZmine 2.38对原始UPLC-HRMS数据进行处理,得到了包含4150个质谱特征的峰列表,其中包含大量的干扰质谱。为有效过滤干扰质谱,提取目标类型化学成分的质谱特征,本研究采用IFS对数据进行处理。以下,以CGAs和GQs质谱特征提取为例,说明IFS提取目标类型化学成分的全过程。

**图1:**
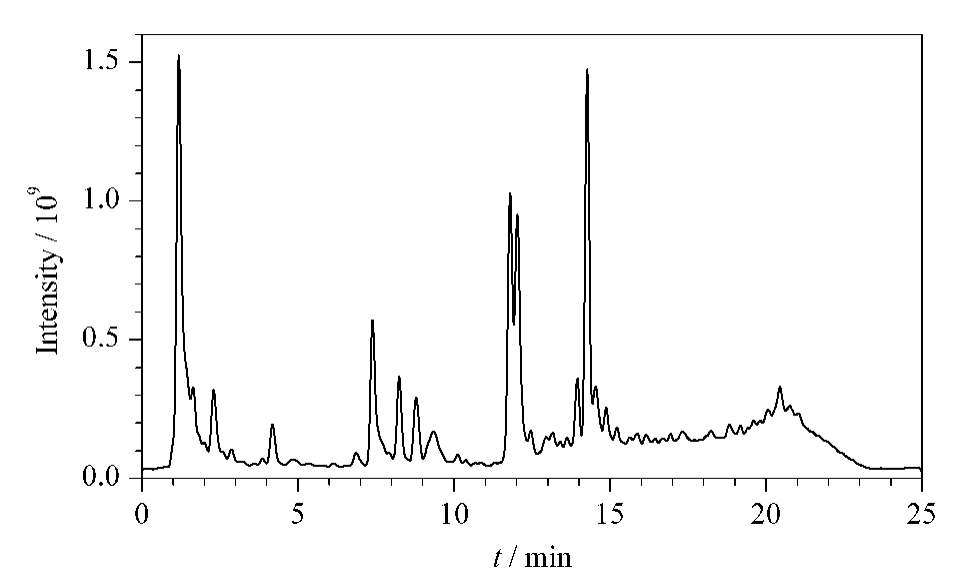
质量控制样品的总离子流色谱图

2.2.1 绿原酸类成分的质谱特征提取

CGAs是奎宁酸(quinic acid, QA)和肉桂酸衍生物酯化形成的一类特殊的酯,最常见的肉桂酸衍生物有对香豆酸、咖啡酸、阿魏酸和没食子酸,其余还有芥子酸、苯丙酸和 3,4,5-三羟基肉桂酸^[15,18,20,21]^。本研究以QA为母体结构,根据酯化反应位置和数量的不同,可以将CGAs的结构分为3种,分别是单取代绿原酸类成分(mono-substituted chlorogenic acid components, Mono-CGAs)、二取代绿原酸类成分(di-substituted chlorogenic acid components, Di-CGAs)以及三取代绿原酸类成分(tri-substituted chlorogenic acid components, Tri-CGAs)。基于取代类型的不同,本研究设定了3个不同的MDF窗口,如表1所示。因此从符合NRF条件的3537个质谱峰中筛选出751个可能的目标成分,如图2a所示。CGAs通常在酯键处产生断裂,并失去肉桂酰基残基,因此产生了*m/z*为191.0551的特征碎片离子^[20]^。由标准品鉴定出的CGAs,包括绿原酸、隐绿原酸和新绿原酸,都包含*m/z*为191.0551的特征碎片离子([QA-H]^-^)。故将*m/z*为191.0551作为CGAs的诊断碎片离子进行进一步筛选,得到了22个符合NRF条件的目标成分(见图2a)。结合MDF、NRF和DFIF,最终筛选出9个目标CGAs成分。

**表1:** 绿原酸类成分质量亏损窗口的设定

Type of CGAs	Mass range/Da	Mass defect range/Da
Mono-CGAs	337-398	0.06-0.12
Di-CGAs	483-604	0.07-0.18
Tri-CGAs	629-810	0.08-0.23

**图2:**
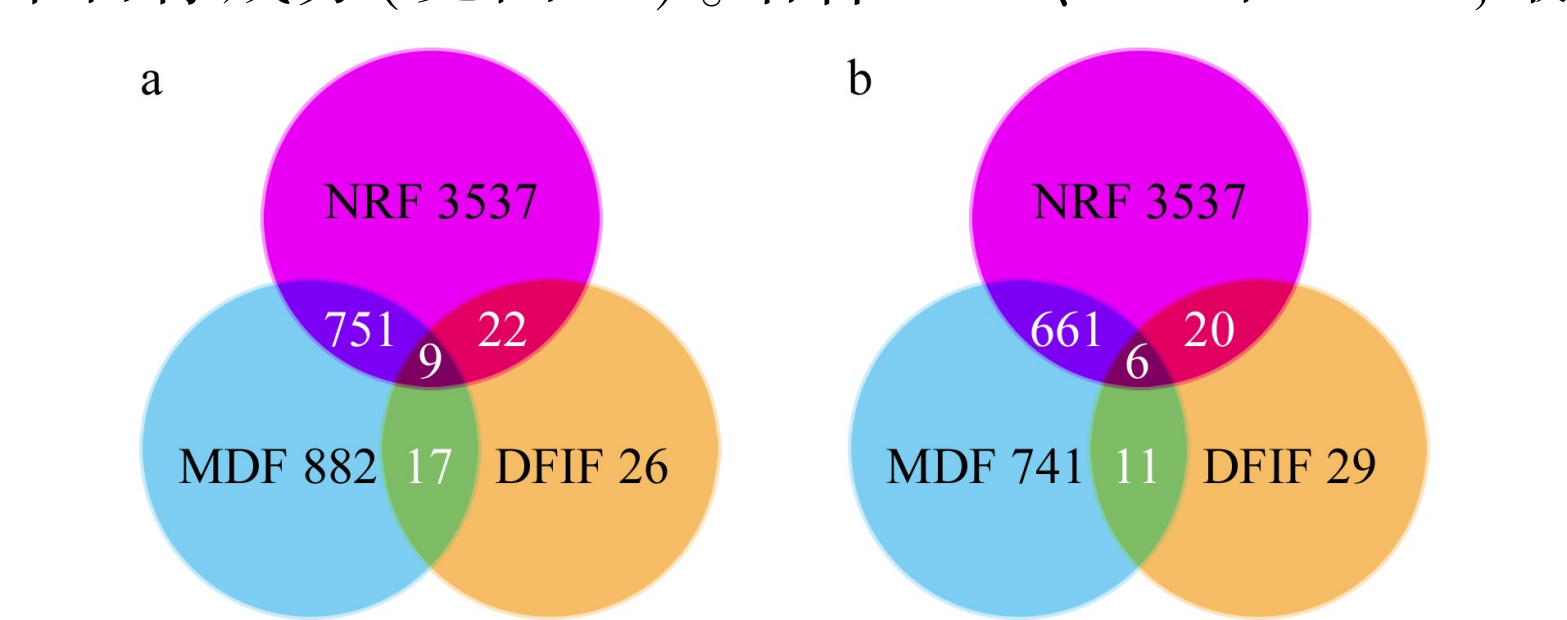
(a)绿原酸类成分和(b)糖基化槲皮素类成分的整合过滤策略筛选结果

2.2.2 糖基化槲皮素类成分质谱特征提取

据文献^[15,22]^报道,糖基化类黄酮中涉及的糖可分为戊糖(如阿拉伯糖和木糖)、脱氧己糖(如鼠李糖)和己糖(如半乳糖和葡萄糖),并且糖基化类黄酮还可以由几种酚酸进行酰基化,例如对香豆酸(*p*-coumaric, *p*-Co)和没食子酸(gallic acid, G)。根据以上信息,可以对GQs的MDF窗口进行设置,如表2所示。共设置了9个不同的MDF窗口,从符合NRF条件的3537个质谱峰中筛选出661个可能的目标成分,如图2b所示。质谱离子化过程中,GQs的酯键容易断裂并失去糖基和/或酰基,从而生成*m/z*为301.0354的特征碎片离子^[15]^。分析利用标准品鉴定出的GQs,包括异槲皮苷、金丝桃苷和芦丁等成分,都包含*m/z*为301.0335的特征碎片离子([quercetin-H]^-^),故将其作为GQs的诊断碎片离子进行后续筛选,得到了符合NRF条件的20个目标成分(见图2b)。结合MDF、NRF和DFIF,最终筛选出6个GQs目标成分。

**表2:** 糖基化槲皮素类成分质量亏损窗口的设定

Type of GQs	Mass range/Da	Mass defect range/Da
Mono-glycoside	433-464	0.07-0.10
Di-glycoside	565-626	0.12-0.16
Tri-glycoside	697-788	0.16-0.21
Galloyl-mono-glycoside	585-616	0.08-0.11
Galloyl-di-glycoside	717-778	0.13-0.17
Galloyl-tri-glycoside	849-940	0.17-0.23
p-Co-mono-glycoside	579-610	0.11-0.14
p-Co-di-glycoside	711-772	0.15-0.19
p-Co-tri-glycoside	843-934	0.19-0.25

由上述结果可知,IFS能够快速有效地去除干扰质谱信息,从而获得目标类型化学成分的质谱特征,该方法在很大程度上提高了化学成分鉴定的准确性和效率^[23]^。现阶段,该方法被应用于中药研究^[24,25]^、茶叶研究^[15]^以及代谢组学研究^[17]^等方面,但在茶树花方面的应用鲜见报道。本研究证实,IFS对于提高茶树花中化学成分鉴定的效率和覆盖面是有效的。

### 2.3 茶树花中化学成分的定性分析

本研究从茶树花中共鉴定出137种化学成分,包括3种生物碱、38种黄酮、31种酚酸及其衍生物、37种儿茶素及其衍生物、18种氨基酸以及10种其他类成分,如表3所示。其中78种化学成分根据标准品进行准确定性,分别为18种氨基酸、13种儿茶素及其衍生物、13种酚酸及其衍生物、24种黄酮、3种生物碱以及7种其他类成分。59种化学成分根据文献以及数据库定性(如TMDB和TPIA),分别为24种儿茶素及其衍生物、18种酚酸及其衍生物、14种黄酮以及3种其他类成分。

**表3:** 茶树花化学成分的定性定量分析结果

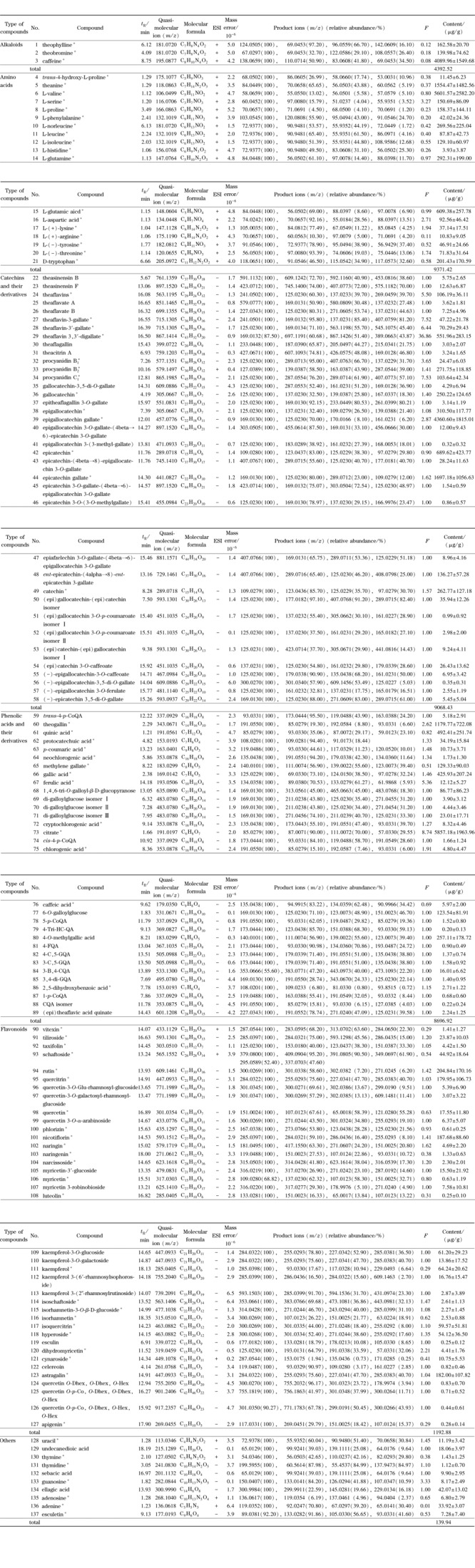

*: identified by standard substances; C: caffeoyI; p-Co: p- coumaroyl; F: feruyl; QA: quinic acid; G, galloyl; B: benzenepropanoyl; Tri-HC: 3,4 ,5- trihlydroxycinamyl; Glu: glucosyl; Hex: hexose; Dhex: deoxyhexose. F: relative correction factor.

### 2.4 茶树花中化学成分的定量分析

本研究采用内标法对茶树花中的化学成分进行定量分析,定量结果如表3所示。137种化学成分中,总含量最高的是氨基酸,约为黄酮类成分总含量的7倍。儿茶素及其衍生物的总含量略低于氨基酸为9068.43 μg/g。本次实验中共鉴定出38种黄酮类成分,约占所鉴定成分总量的28%。在38种黄酮当中,单个成分含量最高的为芦丁,含量为204.84 μg/g。咖啡因(4089.96 μg/g)、L-缬氨酸(5601.57 μg/g)和表没食子儿茶素没食子酸酯(4360.60 μg/g)分别为生物碱、氨基酸和儿茶素及其衍生物中含量最高的成分,含量分别约为芦丁含量的20倍、27倍以及21倍。儿茶素单体相较其他儿茶素及其衍生物的含量更高,总含量占儿茶素及其衍生物总含量的83.5%。除儿茶素单体以外,含量最高的单体成分是茶黄素-3,3'-双没食子酸酯,含量为551.96 μg/g。

## 3 结论

本研究采用UPLC-HRMS技术实现了茶树花中多类化学成分的同时分析检测。在此基础上,利用IFS对目标类型化学成分进行快速靶向识别,提高了茶树花中化学成分定性分析的效率和覆盖面。本研究共鉴定出茶树花中的化学成分137种,并利用内标法进行定量分析,实现了茶树花中化学成分组成和含量的全面分析。研究结果有利于研究者全面了解茶树花中化学成分的富集情况,为茶树花的深度开发和利用提供有价值的信息和数据参考。
